# Quercetin: A Potential Polydynamic Drug

**DOI:** 10.3390/molecules28248141

**Published:** 2023-12-17

**Authors:** Nikitas Georgiou, Margarita Georgia Kakava, Efthymios Alexandros Routsi, Errikos Petsas, Nikolaos Stavridis, Christoforos Freris, Nikoletta Zoupanou, Kalliopi Moschovou, Sofia Kiriakidi, Thomas Mavromoustakos

**Affiliations:** 1 Laboratory of Organic Chemistry, Department of Chemistry, National and Kapodistrian University of Athens, Panepistimiopolis Zografou, 15771 Athens, Greece; nikitage@chem.uoa.gr (N.G.); alexroutsi@chem.uoa.gr (E.A.R.); errpets@chem.uoa.gr (E.P.); nikosstav@chem.uoa.gr (N.S.); nikolzoup@chem.uoa.gr (N.Z.); kmoschovou@chem.uoa.gr (K.M.); sofki@chem.uoa.gr (S.K.); 2 Laboratory of Organic Chemistry and Biochemistry, Department of Chemistry, University of Patras, 26504 Patras, Greece; up1068935@ac.upatras.gr; 3 Center of Excellence for Drug Design and Discovery, National and Kapodistrian University of Athens, 15771 Athens, Greece; 4 Laboratory of Analytical Chemistry, Department of Chemistry, National and Kapodistrian University of Athens, Panepistimiopolis Zografou, 15771 Athens, Greece; xrifre@chem.uoa.gr; 5 Departamento de Quimica Orgánica, Facultade de Quimica, Universidade de Vigo, 36310 Vigo, Spain

**Keywords:** quercetin, flavonoids

## Abstract

The study of natural products as potential drug leads has gained tremendous research interest. Quercetin is one of those natural products. It belongs to the family of flavonoids and, more specifically, flavonols. This review summarizes the beneficial pharmaceutical effects of quercetin, such as its anti-cancer, anti-inflammatory, and antimicrobial properties, which are some of the quercetin effects described in this review. Nevertheless, quercetin shows poor bioavailability and low solubility. For this reason, its encapsulation in macromolecules increases its bioavailability and therefore pharmaceutical efficiency. In this review, a brief description of the different forms of encapsulation of quercetin are described, and new ones are proposed. The beneficial effects of applying new pharmaceutical forms of nanotechnology are outlined.

## 1. Introduction

**Scheme 1:**
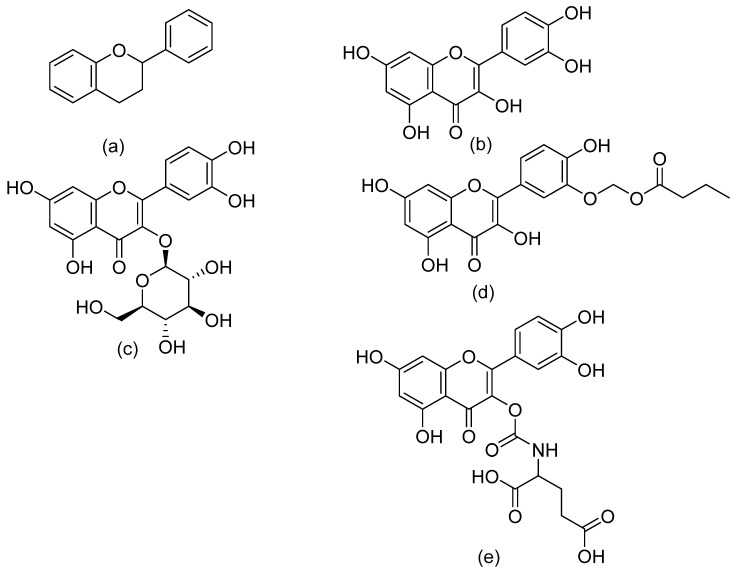
Basic skeleton using Chem Draw of (**a**) 2-phenyl chromane flavonoid (PubChemCID: 94156), (**b**) quercetin (PubChemCID: 5280343), (**c**) isoquercetin (PubChemCID: 5280804), (**d**) quercetin derivative that is effective against MDR cancer cells [1], and (**e**) quercetin-glutamic acid [2].

Flavonoids [1] (Scheme 1a) are a family of organic compounds found mostly in plants and in the food that humans consume. They exert many important biological actions, with favorable antioxidant effects. Flavonoids can be classified into different classes, depending on the substitutes of carbons of the rings [2]. One of these classes is flavonols. Flavonols are flavonoids with a keto group. Flavonols occur in vegetables and fruits such as onions, tomatoes, and apples. One of the most studied flavonols is quercetin (Scheme 1b).

Quercetin [3,4] is an organic compound that belongs to the family of flavonoids, with a wide range of medical properties [5,6]. Some of these include anti-allergy, anti-inflammatory, anticancer, anti-tumor, and antiviral properties as well as cardiovascular protection. It has also been found that quercetin plays a vital role in plants [7]. Specifically, quercetin has antioxidant and antimicrobial activities, and as a result, it contributes to photosynthesis, growth, and seed germination. Moreover, the presence of quercetin in various regions of the brain contributes to combatting against various neurological diseases such as Alzheimer’s and Parkinson’s [8]. Until now, there is no specific treatment for these diseases, but flavonoids—and especially quercetin—have been used for treatment in animal models. 

The skeletal formula of quercetin [9] was acquired using Chem Draw to find some physicochemical and toxicity properties through the SwissADME [10], pkCSM [11], and preADME [12] platforms. This procedure is very important for computational drug design. Some potential biological compounds fail to reach clinical trials due to their unfavorable (ADME) parameters [13,14,15,16,17]. 

Quercetin’s molecular weight is <500 g/mol, its number of hydrogen-bonding donors is less than five, its number of hydrogen-bonding acceptors is less than 10, and its lipophilicity [18] is less than five. As a result, it obeys Lipinski’s Rules of Five. Also, Veber’s Rule [19] is qualified, because the number of rotatable bonds is less than seven. Quercetin has not been predicted to be hepatotoxic, and it has no skin sensitization [20]. Due to the fact that its blood brain barrier index (BBB) [21] is less than one, it is considered as inactive to the central nervous system (CNS). Also, it might be better absorbed from the intestinal tract on oral administration. It has very low solubility in water (about 1 μg/mL) and low bioavailability. Its low bioavailability has led researchers to synthesize various complexes with quercetin engulfed in transfer vehicles. 

The SwissTarget platform was employed in order to assess quercetin’s inhibitory activity. Inhibitory activity has been observed against a plethora of enzymes such as monoamine oxidase A, monoamine oxidase B [22], and thrombin or lipoxygenase [23]. Lipoxygenases belong to the category of oxidoreductases and are widely found in plant organisms, fungi, and animals. Such enzymes are not commonly found in yeasts and bacteria and are not elements of a typical prokaryotic cell. 

Bioavailability is the ability of a compound to be active inside the organism and to enter systemic circulation. Quercetin is lipophilic, with poor water solubility. Therefore, its bioavailability is low, and for that reason, a common strategy to increase its bioavailability is for it to be engulfed in biomolecules and form soluble complexes. 

## 2. Polydynamic Biological Activity of Quercetin

### 2.1. Mental Activity

Quercetin can play a significant role in mental health diseases [24] such as depression and anxiety. Mice studies have demonstrated that some natural products including quercetin possess anxiolytic properties when administered orally. Moreover, they are unlikely to have side effects serious enough to prevent their pharmacological utility, so they could constitute the starting point for the development of more selective anxiolytic agents [25]. Due to its antioxidant activity, quercetin can lower nitric oxide and some other compounds that are vital for these diseases. According to SwissADME, quercetin can inhibit CYP isoenzymes. As a result, it can protect the organism from pathogenic factors. 

### 2.2. Ultraviolet (UV) Activity

One study has shown that quercetin encapsuled with polymer nanoparticles can be efficient for sun protection [26]. In recent years, ultraviolet (UV) radiation has been considered a public threat for health worldwide, as it is responsible for acute and chronic skin diseases, such as burns, premature aging skin, and carcinogenesis. Skin cancer is the most common type of cancer that is diagnosed worldwide. It can cause a high degree of mortality when it develops into its most severe form, that of melanoma. Thus, necessary protection is required during exposure to sunlight. Today, sunscreens are used to protect us from early photoaging, photosensitivity, skin cancer, and free radical damage. The main goal of sunscreens is to protect the human skin from UVA and UVB radiation. Recent studies have shown that compounds from natural plants may act as sun protectors [27]. Quercetin is one of those natural products that can reduce the damage from UV radiation.

### 2.3. Antiviral Activity

**Scheme 2:**
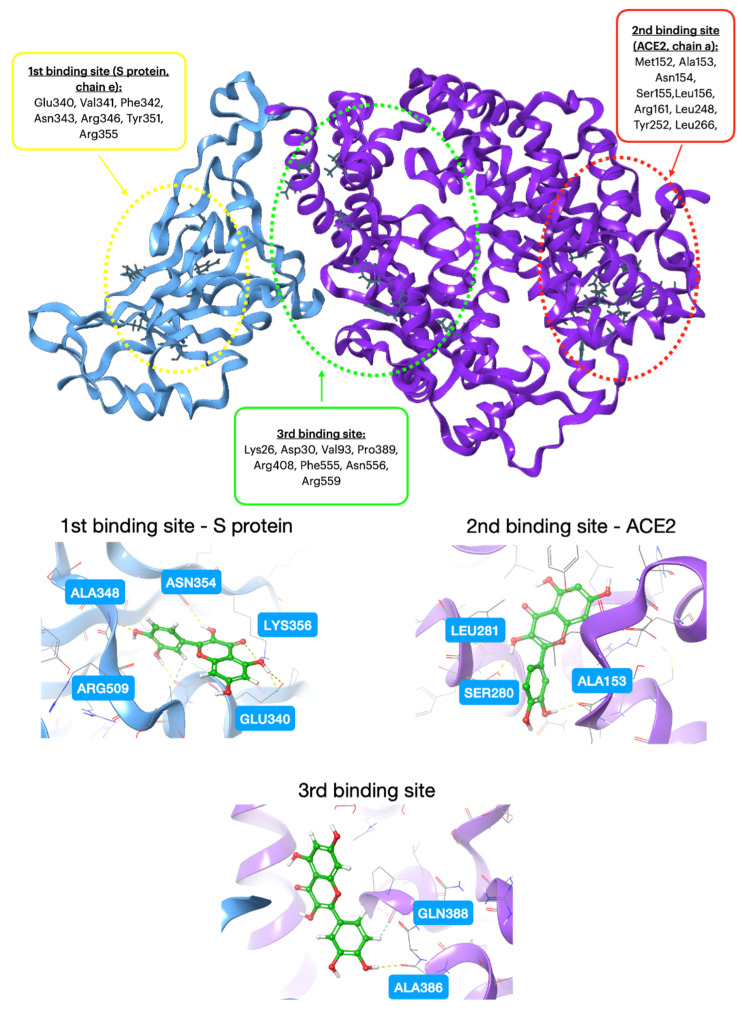
Three-dimensional (3D) structure of spike protein bound to ACE2 (PDB ID: 6M0J). The dotted circle identifies the amino acids that constitute the respective binding sites for the Induced Fit Docking (IFD) experiments (**above**). The interactions were developed from the IFD experiments for quercetin with spike proteins of SARS-CoV-2 from the three studied binding sites (**bottom**). This image was sketched using Maestro software Version 10.2.

Viral diseases are still a problem even after the discovery and use of antiviral drugs for more than 60 years now, due to the toxicity of some new antiviral preparations and the development of resistant viral strains. The human immunodeficiency virus (HIV) [28] is another disease that started to spread throughout the world. HIV has two categories, HIV type 1 and HIV type 2 (HIV-2). HIV was first recognized in 1981 in the USA. The origin of this virus is primate lentiviruses, which exist in chimpanzees. These animals became the host of the virus, which is then transmitted to humans after mutations [29]. Quercetin and isoquercetin (Scheme 1c) have antiviral activities against many types of viruses, including human immunodeficiency virus. Many scientists have suggested quercetin as an antiviral drug due to the fact that it can inhibit the first stages of the virus infection. Quercetin has also been found to exert important pharmacological activity against several other viruses [30]. One such activity is against the Severe Acute Respiratory Syndrome Coronavirus 2 (SARS-CoV-2) [31,32], which recently emerged as a global threat to human health. It is the main cause of the COVID-19 pandemic that caused more than 6,000,000 deaths worldwide. Quercetin has been found to be able to interfere with SARS-CoV-2 and reduce the inflammation provoked by COVID-19 (Scheme 2). Also, blood tests have indicated that quercetin can reduce the time during which the molecular test appears positive by reducing the viral charge [33].

### 2.4. Anticancer Activity

Cancer is a serious disease that hurts many developed and developing countries. There are more than 100 types of cancer. Most often, a specific type of cancer is characterized by the type of cell in which it is formed. The most basic types of cancer are carcinoma, sarcoma, leukemia, lymphoma, multiple myeloma, melanoma, and brain and spinal cord tumors [34].

Quercetin has been found through in vitro experiments to exhibit anti-tumor activity against prostate [35,36], liver [37], breast [38], and pancreatic [39] cancer and melanoma [40]. Quercetin’s anticancer effects on hepatocellular carcinoma [41] have been studied not only in vitro but also in vivo. Although the exact mechanism of action remains elusive, quercetin’s anticancer effect may arise by regulating some enzymatic activities or also by modulating oxidative stress and some cellular pathways. 

Chemoprevention involves treating cancer before it becomes aggressive, but it does have its downsides. This approach can potentially lead to side effects and toxicity. Quercetin has demonstrated synergistic effects in addressing tumors with multidrug resistance by blocking the expulsion of drugs facilitated by transporter proteins. It is used as a low-toxicity medicine. Studies have shown that the anticancer activity of quercetin can be improved by encapsulating quercetin inside nanoparticles. In vitro and in vivo studies have shown successful tumor treatment using quercetin nano-formulations. These approaches can reduce side effects. Some examples include polymeric nanoparticles, non-responsive polymeric nanoparticles, and stimuli-responsive polymeric nanoparticles. There are also examples of inorganic nanoparticles with quercetin—specifically, silica nanoparticles, gold nanoparticles, and metal oxide nanoparticles [42]. 

### 2.5. Anti-Inflammatory Activity

Moreover, quercetin has been shown to exert anti-inflammatory activity. Inflammation is a multifactorial and complex biological response of body tissues to harmful stimuli, so as to restore the organism to homeostatic balance. Inflammation is found in some areas of the body and refers to the tissues of an organ or a tissue or a whole organ, etc. (e.g., arthritis, tendonitis, stomatitis, peritonitis, etc.). Rheumatoid arthritis is an example of an autoimmune inflammatory disease [43]. This disease affects more women than men, and it was discovered many years ago. The symptoms of this disease are stiffness and swelling that appear in the feet, fingers, and toes. In vitro [44] studies have shown that quercetin may be a good drug candidate for the treatment of this disease, because it can inhibit neutrophil activity. It can also inhibit the activation of NLRP3 inflammasomes [31]. Lipoxygenases (LOXs) are a group of monomeric oxidant metalloproteins, containing a non-heme-coordinated iron atom (non-heme ion Fe) [45,46]. Moreover, several in vitro experiments have shown that quercetin can inhibit soybean lipoxygenase [3], which is involved in inflammation.

### 2.6. Neurological Activity

Alzheimer′s disease (AD) [47] is a fatal complex neurodegenerative disease that affects more than 24 million people worldwide [48]. The disease is characterized by multiple pathological features and is clinically associated with cognitive impairment, language loss capacity, and dementia. Current treatment options include results with moderate improvement of memory and cognitive function; however, they do not prevent progressive neurodegeneration. Multifunctional compounds capable of simultaneously interacting with the ingredients of many pathologies have been considered as a solution and are being researched for the treatment of complex pathologies of neurodegenerative diseases [49,50]. Quercetin is one of these compounds that can be used against Alzheimer’s disease due to the fact that it has a neuroprotective effect against oxidative stress [51]. 

### 2.7. Antioxidant Activity

When found in moderate concentrations, the active forms of oxygen (reactive oxygen species, ROS) participate in the normal processes of the organism, but their production in large concentrations leads to oxidative stress, disrupting the organism’s cellular oxidation balance [52,52,53]. Antioxidants are substances that can protect cells against oxidation and the effects of free radicals, because they annihilate these radicals from the medium. They also constrain oxidation by oxidizing themselves. Antioxidants suppress various harmful activities of ROS, so they are used to prevent or treat such diseases. One health problem that oxidative stress is associated with is obesity. Obesity is one of the major health problems in the world, and it leads to increased amounts of fat cells. It is characterized by the overproduction of reactive oxygen stress. Quercetin, along with other natural products, has been shown to exert beneficial effects against obesity through different molecular pathways. In vivo experiments using obese rats have been shown to lose weight after treating with quercetin [54]. 

Moreover, Jose Angel Maranon Maroto proved that a combination of the polyphenols resveratrol, quercetin, and catechin has synergic antioxidant power. Polyphenolic compounds of natural origin are recognized as antioxidant agents, which act as free radical scavengers. Resveratrol is a polyphenolic compound that is present mostly in seeds and in the skin of grapes and other plant products [55]. 

### 2.8. Anti-Cardiovascular Disease

Heart diseases or cardiovascular diseases are those diseases that involve the heart or blood vessels (arteries and veins) [56]. Though the term technically it refers to any disease affecting the cardiovascular system, it is usually used to refer to those related to atherosclerosis (arterial diseases). These diseases present similar causes, mechanisms, and treatments. Most countries face high and increasing rates of cardiovascular diseases. It has been shown that they affect adolescents and kids, and for this reason, prevention against them is mandatory from childhood. When heart problems are diagnosed, the underlying cause (atherosclerosis) is usually quite advanced. Therefore, more emphasis is placed on the prevention of atherosclerosis through the modification of risk factors, such as healthy eating, exercise, and avoiding smoking. The protective effects of quercetin against cardiovascular diseases include the reduction of blood pressure and arterial pressure. Hypertension is the most common cause of cardiovascular diseases, such as in cardiac hypertrophy, responsible for abnormal cardiac growth, which leads to arrhythmia, myocardial infraction, and heart failure [57]. Many people currently suffer from hypertension, an alerting sign that pharmacological and natural interventions are needed in order to decrease blood pressure and inhibit various biochemical pathways that are involved in cardiovascular diseases. Studies have demonstrated that quercetin can decrease blood pressure via multiple mechanisms like inhibiting protein kinase C (PKC), a family of protein kinase enzymes implicated in governing heart failure [58], decreasing oxidative stress, inhibiting angiotensin, converting enzyme activity, or even modulating cell signaling and gene expression [59].

### 2.9. Skin Sensitivity

Numerous individuals experience skin wounds, which can be either chronic or temporary and may affect a substantial area of the skin. Healing processes typically fall into three categories: primary healing (also known as healing by first intention), which takes place within 12 to 24 h after the wound forms; secondary healing (or healing by second intention), observed in wounds with significant loss of soft tissue; and the healing of superficial wounds, such as those seen in superficial burns and abrasions, involving the epithelium and the papillary part of the dermis. Natural products, especially those from plants, are a new strategy for wound healing. Quercetin is used for the treatment for wounds because, as outlined, it shows anti-inflammatory activity. *Rubusniveus* [60] is one of the species against which quercetin was found to have some wound-healing activity [61].

There are a lot of examples of the biological activity of quercetin occurring in in vivo experiments. One example is in the species of *Bergia ammannioides* [62], against which quercetin was found to have antioxidant and anti-inflammatory abilities. Secondly, in *Melilotus officinalis* and in *Lespedeza capitata* [63], quercetin was found to increase the HaCaT human keratinocytes. Furthermore, in *Martynia annua* and *Tephrosia purpurea* [64], quercetin was found to have antioxidant activity. Also, quercetin has protection against endotoxin-induced inflammatory response [65], surgical-induced osteoarthritis [66], LPS-induced oxidative stress and inflammation [67], LPS/interferon c-induced nitric oxide production [68], TNF-α induced inflammation [69], and CCl4-induced inflammation [70].

### 2.10. Anti-Tuberculosis

Tuberculosis is a fatal infectious disease caused by the Mycobacterium tuberculosis (*Mycobacterium tuberculosis*). Despite the availability of effective treatment, tuberculosis is responsible for a million deaths worldwide per year. The bacterium has developed a resistance to the drugs on the market, and so the need arises to find other therapeutic compounds [71,72]. Quercetin can be a good inhibitor for the bacterium [73]. This was found through in vitro antituberculosis bioassays.

### 2.11. Antidiabetic Activity

Insulin is a protein hormone that is necessary for the maintenance of normal blood glucose levels, either by increasing peripheral glucose uptake or by suppressing the production of hepatic glucose [74]. Quercetin might be a promising candidate that acts in many targets of diabetes, and it can regulate many pathways [75,76]. Furthermore, co-crystals comprised of quercetin and antidiabetic agents like metformin and DPP-IV inhibitors have been demonstrated to treat diabetes mellitus (DM) by reducing blood glucose levels and improving glucose tolerance. DM is a chronic disease that is diagnosed as a result of elevated blood glucose levels caused by inadequate insulin secretion, defective insulin action, or both [77,78].

### 2.12. Antimalaria Activity

Malaria is one of the most threatening tropical diseases that leads to millions of deaths every year. Almost all fatal cases are caused by *Plasmodium falciparum* and its strains, which have developed resistance to the drugs in circulation. Therefore, a need has arisen for new active compounds for the treatment of this disease. Quercetin is a potential antimalaria drug, as proven through in vitro experiments [79].

### 2.13. Antichagas Activity

Chagas disease (CD) [80] is a disease that many scientists ignore, and its main bacteria is the *Trypanosoma cruzi* (TC). This disease appears mainly in Central and North America, but in recent decades, the number of CD cases has been increasing in other countries, such as in the south of the United States of America, in Canada, in the Western Mediterranean, and in the Western Pacific. It is estimated that about 6 to 7 million people are potentially infected by TC, which causes about 20,000 deaths per year and is the leading cause of infectious myocarditis. Quercetin and other flavonol derivatives can be antitrypanosomal candidates, showing IC_50_s of 0.6, 0.7, 0.8, and 1.0 µg/mL [81].

### 2.14. Antifungal Activity

Fungicides have often been observed to pose a risk to human health and can be harmful to the environment. Thus, there is a need to find alternative solutions to deal with fungi, with natural compounds that will not affect either human health or the environment. The *Candida parapsilosis* species is composed of three other species, i.e., *C. parapsilosis sensu lato*, *C. orthopsilosis,* and *C. metapsilosis.* These species are found in vegetables and fruits, and they are known to cause infections worldwide. Quercetin has been shown to have antifungal activities through the determination of its minimum inhibitory concentration (MIC) [82].

### 2.15. Combination of Quercetin with Other Drugs

There are several examples whereby quercetin and its derivatives have been combined with other compounds with biological interest and activity. One of these examples is sickle cell disease. Sickle cell disease and its variants constitute the most common blood disorders, affecting millions of individuals worldwide. Until now, there has been no treatment for this disease, and there is no acute method for prevention [83]. Another example is Fragile X Syndrome. This disease is the most common one implicated in intellectual disability. Someone who suffers from this disease has many serious medical problems [84]. Examples of the use of quercetin with other drugs will be described in the Section 3.

### 2.16. Anti-Rhinitis Activity

Acute rhinitis is one of the most common inflammatory diseases in Western countries. The major symptoms include nasal obstruction and nasal secretions. In the evolution of the disease, a frequent complication is acute rhinosinusitis, which can progress to chronic rhinosinusitis and then to intracranial complications, meaning that it is necessary to treat the disease as soon as possible. The cause of rhinosinusitis lies in the secretion of pro-inflammatory cytokines, key factors in initiating inflammation, consequently leading to local edema and swelling of the mucosa and an increase in nasal and sinus secretions. Quercetin has proven its antioxidant and anti-inflammatory properties against rhinosinusitis, both in rats and humans, by inhibiting the release of chemical mediators, such as histamines and leukotrienes, and reacting with relative oxygen species (ROS), which are also involved in rhinosinusitis [85,86].

### 2.17. Antidrug Resistance

Multidrug resistance (MDR) is defined as the ability of cancer cells to survive treatment with a variety of anticancer drugs, similar to the concept commonly applied to antibiotic treatment [87]. MDR is responsible for over 90% of deaths in cancer patients receiving chemotherapeutics or targeted drugs. Derivatives of quercetin (Scheme 1d) have been proven to be possible candidates in treating MDR cancer, as well as viral infections in humans [88].

Skeletal muscles are tissues that are involved not only in mobility and movement but also in glucose and lipid metabolism. Muscle atrophy is the loss of skeletal muscle mass due to increased myofibrillar protein degradation. It occurs under various circumstances such as injury and during side effects of pharmaceutical therapy and aging. Muscle atrophy causes falls, and therefore, it has become a serious problem, especially in aging society. Quercetin glucosides are proven to be perfect candidates in the treatment of muscle atrophy, since they play an important role in the downregulation of myostatin signaling via phosphorylation, a possible mechanism responsible for the inhibitory effects of quercetin glucosides [89,90]. 

**Scheme 3:**
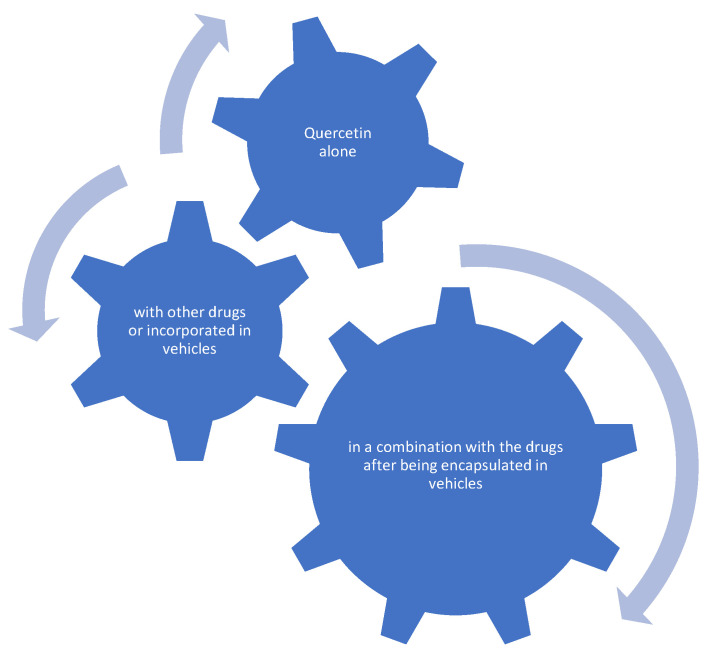
Quercetin alone bears low bioavailability and solubility. In an attempt to increase both its bioavailability and solubility, it was encapsulated in vehicles using nanotechnology. In addition, it is administered with other drugs. We propose that it can be administered with drugs but also given as an encapsulated molecule in vehicles.

All in all, quercetin is commercially available and is one of the most common natural products. Natural products have become more popular, and they have started to be used as lead compounds in medicine. They have a lot of advantages in contrast to common drugs. For instance, they have less side effects. In addition, flavonoids play a significant role in humans and plants. Efforts that have been made to increase the bioavailability and solubility of quercetin are outlined in the Results and Discussion section (Section 3). Basically, vehicles have been used, and quercetin is also administered with other drugs. We propose that a mixture of quercetin engulfed in vehicles along with other drugs should be tried (Scheme 3).

## 3. Results and Discussion

Quercetin interacts with various biomolecules, including cyclodextrin [91,92,93,94,95]. Cyclodextrins are macromolecules that are commonly employed in the food and pharmaceutical industries, serving diverse purposes within these fields [96]. 

**Scheme 4:**
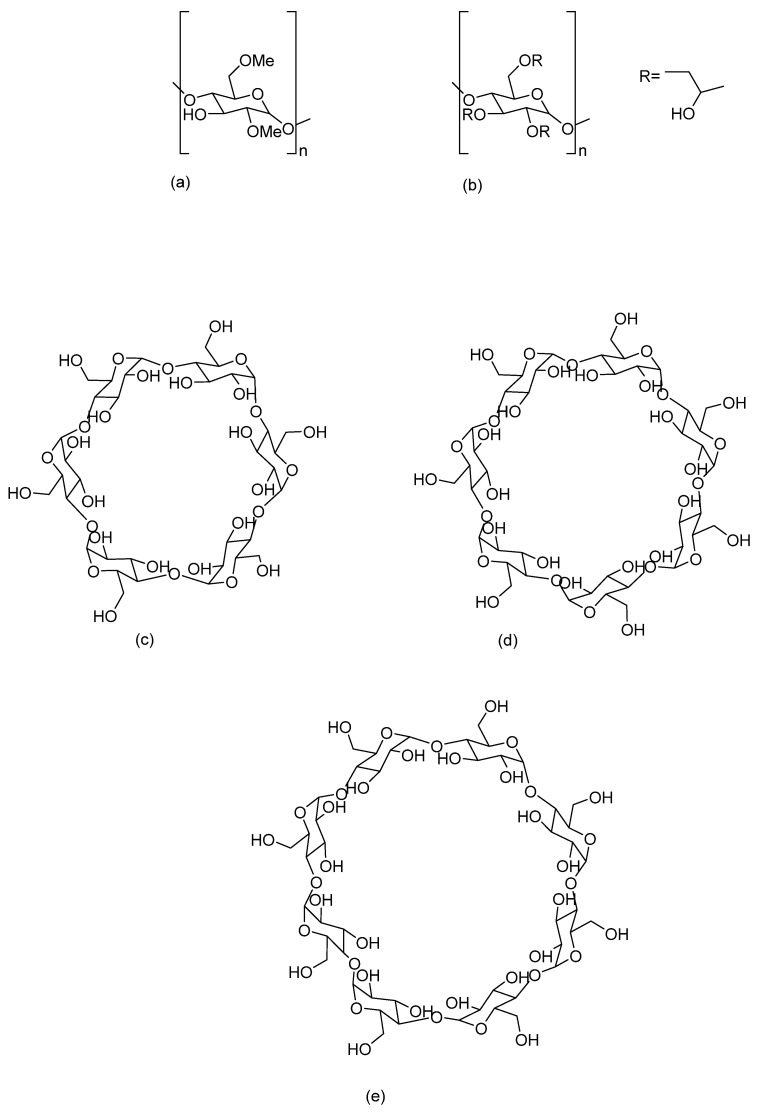
Structure sketched in ChemDraw of (**a**) Me-β-CD, (**b**) 2HP-β-CD, (**c**) α-cyclodextrin, and (**d**) β-cyclodextrin and (**e**) γ-cyclodextrin.

Cyclodextrins [97,98] are cyclic macromolecules composed of glucopyranose units [99,100]. The outer surface of cyclodextrins is hydrophilic, and the inner is hydrophobic [101]. They are soluble in water. The most common cyclodextrins are composed of six, seven, and eight glucopyranose units, which are α-, β-, and γ-cyclodextrins (Scheme 4). Nevertheless, nowadays, a plethora of cyclodextrin derivatives have been synthesized that are comprised of less than six glucopyranose units. Larger cyclodextrins have also been achieved with more than eight glucopyranose units. Indeed, a wide range of cyclodextrins can be generated by employing different substitutes, leading to a diverse array of these molecules, with varied properties and applications. CDs can improve the bioavailability of drugs [99,102]. They play a vital role in computational drug design, and there are a lot of medicines with CD-drug complexes that are already in commercial use [103]. One example of CD-drug complexes is those with curcumin [101,104,105].

**Scheme 5:**
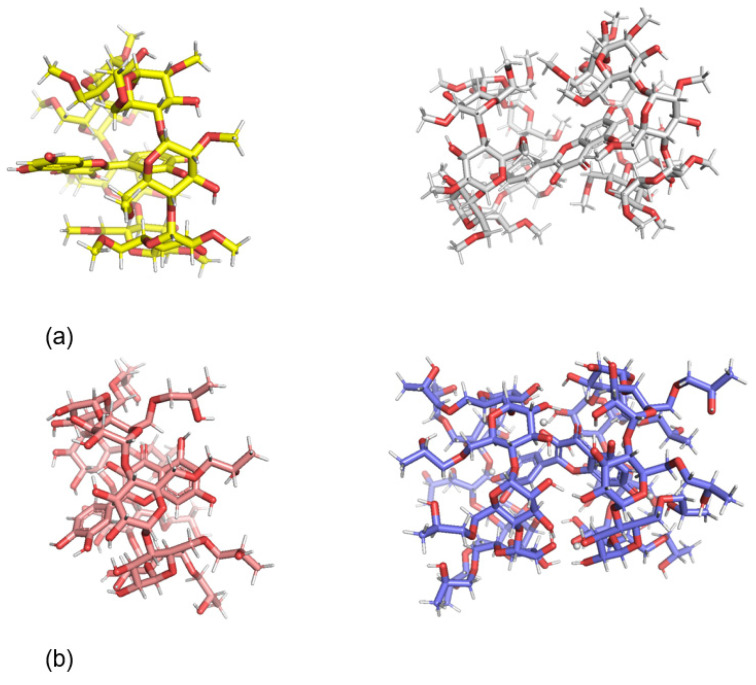
Calculated minimum structure of complex (yellow and pink, right) and dimeric assembly (white and blue, left): (**a**) QUE with 2,6Μe-β-CD and (**b**) QUE with 2HP-β-CD. This image was sketched in Pymol.

The synthesis of these complexes was performed by freeze-drying [106]. Moreover, 2D DOSY NMR experiments have shown the complexation between quercetin and 2HP-β-CD and 2,6Me-β-CD (Scheme 5). Quantum chemistry studies employing Density Functional Theory have shown that the binding between quercetin and dimeric assemblies of 2HP-β-CD and 2,6Me-β-CD (Scheme 5) cyclodextrins is relatively weak, leading to facile quercetin entrance and exit from the CD vehicle [92]. Fluorescence spectroscopy and molecular Dynamics experiments have shown that these dimeric assemblies remain stable. The quercetin-CD complex is stabilized through hydrophobic interactions. However, the 2HP-β-CD_2_ dimeric assembly is more stable than 2,6Me-β-CD_2,_ due to stronger binding [91]. The weak quercetin–CD binding allows quercetin to remain available at the intended target site, facilitating selective and safe action. Solubility experiments conducted on these complexes revealed an increase in the solubility of quercetin when encapsulated within cyclodextrins, particularly noticeable at pH 6.8.

Furthermore, the systematic exploration and analysis of quercetin-based compounds that are chemically modified through the incorporation of amino acids was examined. In silico experiments, in vitro assays in different cancer cells, and NMR spectroscopy were used to reveal the interactions between these analogues with the Bcl-xl protein. It was shown that these analogues bind strongly in the protein and remain stable in the active center. Specifically, the conjugation of quercetin with amino acids, particularly Que-Glu (Scheme 1e), enhances quercetin’s inhibitory effects on prostate cancer cells. This approach could offer a promising strategy to improve the therapeutic efficacy of these compounds [107].

One other approach to increase the bioavailability of quercetin is the use of nanoparticles [8,42]. The encapsulation of quercetin in nanoparticles has shown high solubility. This has led to the treatment of various diseases. The size of quercetin nanostructures is between 20 and 50 nm. Studies have shown that quercetin has more antioxidant activity inside nanostructures than free quercetin does. Even though there are treatments with quercetin against cancer, there is low availability of quercetin-involved nanoparticles to treat neurodegenerative diseases [108,109,110,111,112].

**Scheme 6:**
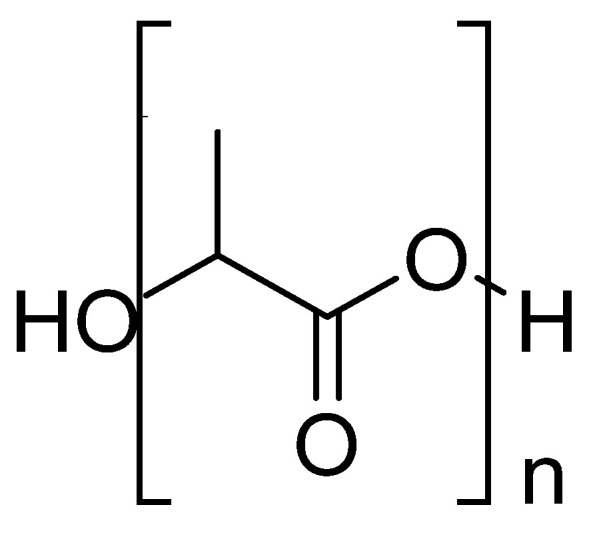
Structure of Poly (D,L-lactide) sketched in ChemDraw.

One other biomolecule that is used for the encapsulation of drugs is poly-d,l-lactide (PLA) (Scheme 6). PLA is often used to form complexes with other molecules due to its high hydrophobicity, biodegradability, and low toxicity. In vitro experiments and fluorescence experiments showed that the antioxidant activity of quercetin was retained inside the polymer. This may lead to the development of nanomedicine and to an antioxidant drug [113]. 

There are several examples in which quercetin and its derivatives are combined with other compounds with biological interest and activity (Table 1). 

Maroto et al. have developed a combination of resveratrol, quercetin, and catechin polyphenols in such proportions that it has a synergistic antioxidant power. They have shown that the preferred embodiment is the combination of antioxidants comprising resveratrol: quercetin: catechin in a 1:1:2 or 1:1:5 molar ratio. The results of the TOSC (Total Oxidant Scavenging Capacity) test showed that it was possible to obtain a potent antioxidant effect without the need to ingest large amounts of resveratrol or the other individual antioxidants, which is advantageous in order to minimize possible risks of side effects. The preferred combination is resveratrol: quercetin: catechin in a 1:1:2 molar ratio. The combination of resveratrol, quercetin, and catechin polyphenols can be used in different pharmaceutical forms, both solid and semi-solid, and these can be included in a variety of pharmaceutical, cosmetic, and foodstuff formulations [135].

**Scheme 7:**
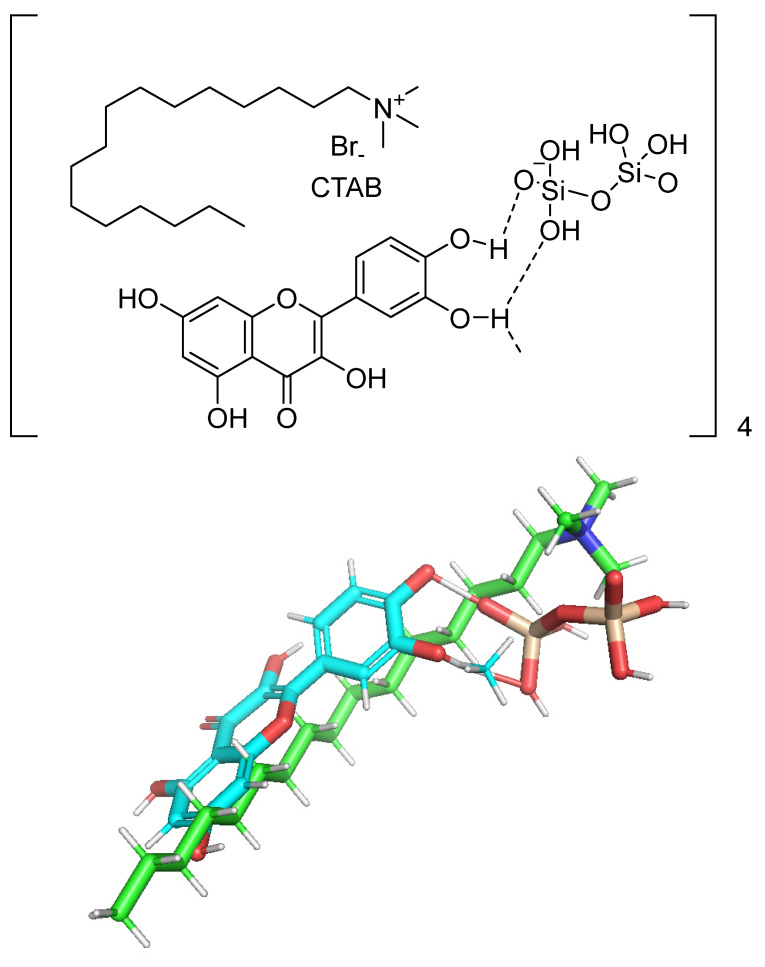
Structure of encapsulated quercetin (blue) inside CTAB nanomaterial (green) in 2D and 3D. This image was sketched in ChemDraw and Pymol.

In another study, it was found that quercetin can be encapsulated in nanoparticles. Specifically, quercetin and silica nanogels were synthesized by aging and drying. The encapsulation was performed in PEGylated and CTAB-modified polymer nanomaterials (Scheme 7). An IR experiment confirmed the stability of quercetin inside the nanoparticles. The antioxidant activity of quercetin was tested in Cu(II), which is a metal that can induce oxidative stress. The entrapment of quercetin within nanoparticles demonstrated its ability to release its contents when exposed to conditions of Cu(II)-induced oxidative stress in neuronal and glial cultures. Such research works aim toward the development of flavonoids in nanomedicine and toward the treatment of Cu(II)-induced oxidative stress in neurodegenerative diseases [136].

As mentioned before, quercetin is being extensively studied as a potential cancer treatment because of its notable characteristics, such as its ability to regulate crucial molecular pathways linked to apoptosis and its effectiveness in inhibiting drug efflux in multidrug-resistant tumors. To address the limitation of poor bioavailability, new formulations leveraging nanotechnology have emerged as a promising solution. Recent in vitro and in vivo studies have showcased successful tumor treatments using nano-formulations loaded with quercetin across various cancer models. These formulations exhibit high quercetin loading percentages in polymeric, lipid, and inorganic nanoparticles. Additionally, the co-delivery of different therapeutic agents has emerged as a promising strategy to elicit synergistic effects. Notably, quercetin has been shown to downregulate membrane transporter proteins, leading to increased intracellular concentrations of other chemotherapeutic compounds, and ultimately improving therapeutic outcomes. These studies are promising for the development and the augmentation of a new series of anti-tumor drugs [42].

**Scheme 8:**
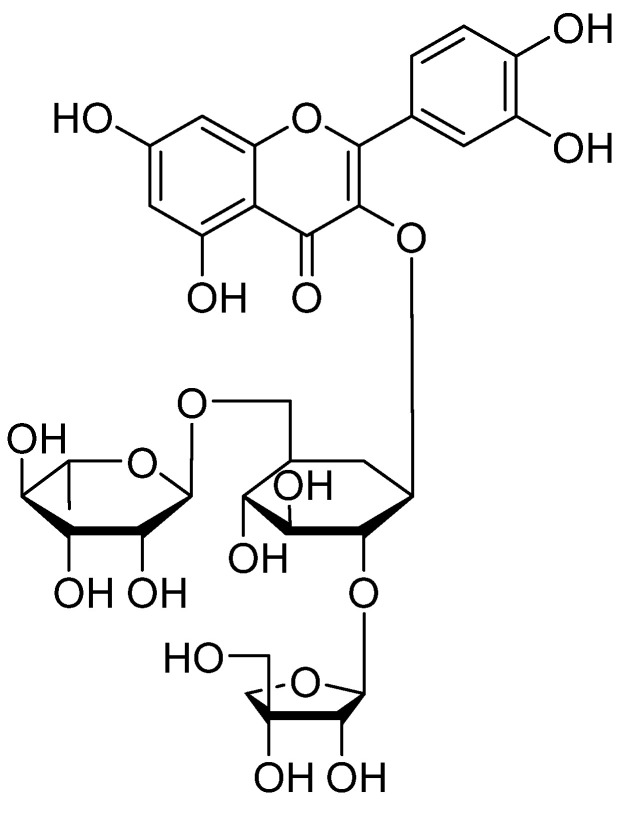
A quercetin-sugar derivative that has been used for treating neurological disorders sketched in ChemDraw.

Additionally, quercetin-sugar derivatives, which are depicted in Scheme 8, have been synthesized and used for the prevention and treatment of diseases related to the 5HT_1A_ receptor by inhibiting it, or neuron cell damages, including drug or alcohol dependence, sleep disorders, panic state, delaying senility, and improving learning and memory [137].

Another study showed that quercetin encapsulated in liposomes can be a candidate for the treatment of ischemia [138]. Liposomes can be used as drug carriers like cyclodextrins. Specifically, the synthesis of quercetin with liposomes was conducted with a thin-film hydration method. In vivo experiments with rats revealed a potent antioxidant activity of quercetin with this nano-formulation.

In the case of flavonoids, solvents play a leading role in their activity. The solvent seems to have an effect on hydrogen bonding through the available donor-acceptor sites in the flavonoid. Based on this, many studies focus on the solubility of flavonoids, especially quercetin, in different organic solvents.

**Scheme 9:**
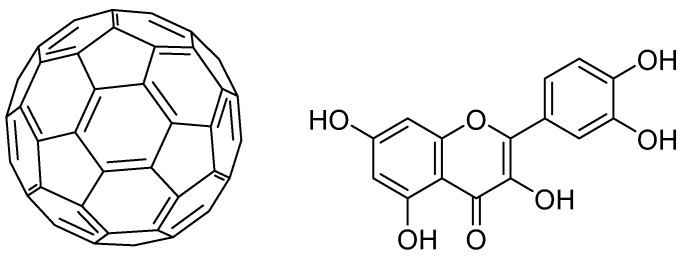
C60 fullerene with quercetin sketched in ChemDraw.

Previous studies have shown that C60 fullerene leads to moderate toxicity because of its low water solubility. This may be harmful to aquatic organisms. One effective way to prevent this is the combination of C60-quercetin solutions (Scheme 9). As a result, the solubilization of C60 with quercetin leads to more biodegradable materials [139]. In recent years, scientists have achieved the synthesis of novel C60 fullerene-flavone derivatives, starting from quercetin, via cyclopropanation (Bingel reaction) of C60. The products of this reaction have antioxidant activity and may be used as novel drug leads [140].

In 2023, Das Saha et al. [141] examined the anticancer potential of quercetin and 5-fluorouracil-encapsulated (5-Fu) chitosan nanoparticles, since chitosan has been intensively investigated and used as a carrier in polymeric nanoparticles for drug delivery in both in vitro and in vivo models [142]. 5-Fu is already a chemotherapeutic drug approved by the FDA for treating various types of cancers [143], and quercetin has the ability to express its anticarcinogenic properties via the modification of intracellular signal transduction and the inhibition of cancer-activating enzymes [144]. These nanoparticles were synthesized using ionic gelation methods and were tested against cancer cell lines HepG2 (liver cancer), HCT116 (colorectal carcinoma), and HeLa (cervical cancer) and the normal cell line Hek293 (kidney cells). The results of the performed MTT assays indicated a higher cytotoxic potential of CS-5Fu-QCT nanoparticles in HCT116 cancer cells and no toxicity on the tested normal kidney cells compared to quercetin alone, meaning that this type of NP may be a very effective anticancer agent against colorectal carcinoma, with minimum to no side effects. The CS-5Fu-QCT NPs’ possible mechanism of action works by causing G0/G1 phase cell-cycle arrest in HCT116 cells and altering the expression of pivotal proteins in the p53/p21 pathway, thus initiating cell apoptosis [141].

The field of nanotechnology, specifically nanomedicine, is an alternative and promising tool in the biomedical sciences. Nanomaterials, nanotubes, and nanoparticles offer a new perspective, and their use and application as carriers can contribute substantially to disease diagnosis, treatment, and monitoring [145,146,147,148,149,150]. The characteristics of these materials, such as their biocompatibility and economic viability, make them satisfactory carriers applied to multiple therapeutic and diagnostic agents [151,152]. Researchers who are motivated by the possibilities of nanomaterial, have found the effective interaction of a nanostructure and quercetin, and particularly the incorporation of a hybrid nanostructure with quercetin-coated titanate nanotube [153]. Studies report that a hybrid nanostructure, especially metal-based (High-Z elements) hybrid nanoparticles (MHNs), noble metals, and organic materials, offer an improvement in the efficacy of radiation therapy and have demonstrated cytotoxicity against tumor cells [154]. Moreover, there is evidence demonstrating that despite the unique and easy modification of a titanate nanostructure, the connection and incorporation of quercetin on it does not alter the morphology of the nanostructure, and its tubular structure is preserved [153]. Due to these facts, the investigation and development of incorporated molecules of quercetin in sodium (NaTNT) and zinc (ZnTNT) titanate nanotubes could interfere in cell proliferation and may be a powerful tool in a medical revolution.

**Scheme 10:**
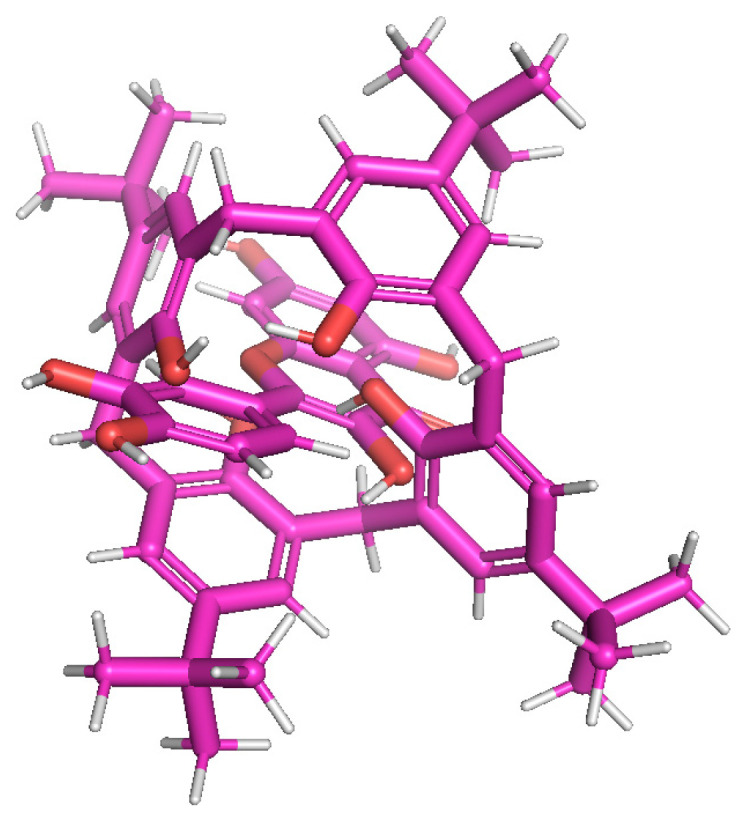
Calixarene interacting with quercetin (purple with red) through DFT methodology. Sketched in Pymol.

**Scheme 11:**
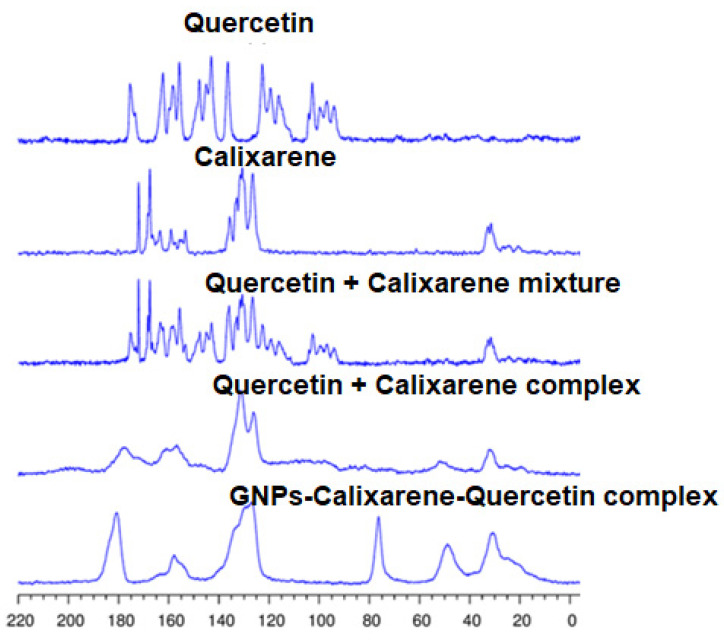
1H−13C CPMAS NMR spectra of quercetin, calixarene mixture, quercetin, and calixarene complex and GNP-calixarene-quercetin complex sketched in TopSpin 4.2.0. software.

Finally, the complexation of quercetin with the calixarene supramolecule was conducted (Scheme 10). To examine this complexation, several analytical techniques were used such as FTIR Analysis, Dynamic and Electrophoretic Light Scattering (DLS), Differential Scanning Calorimetry (DSC), and High-Resolution Transmission Electron Microscopy (HR-TEM). At first, quercetin was enclosed within a calixarene. This resulted in a significant increase of 62,000 times in aqueous solubility. Through solid-state NMR (Scheme 11) and in vitro and in silico experiments, it was found that the complex effectively hindered the growth of tumors, leading to a decrease in tumor volume. Next, a gold nanoparticle core was adorned with calixarene hosts to non-covalently accommodate nanoparticles (GNPs). The nanocarrier loaded with the NP quercetin significantly increased the cytotoxicity (more than 50-fold) compared to the original NP in colon cancer, and it also modified its cell membrane transport mechanism. This enhanced the tumor-targeting properties achieved through this innovative combination, shedding light on a promising avenue for advanced cancer therapies [155].

In Table 2, it is shown all the techniques that they were used for the encapsulation of quercetin in macromolecules.

## 4. Conclusions

**Scheme 12:**
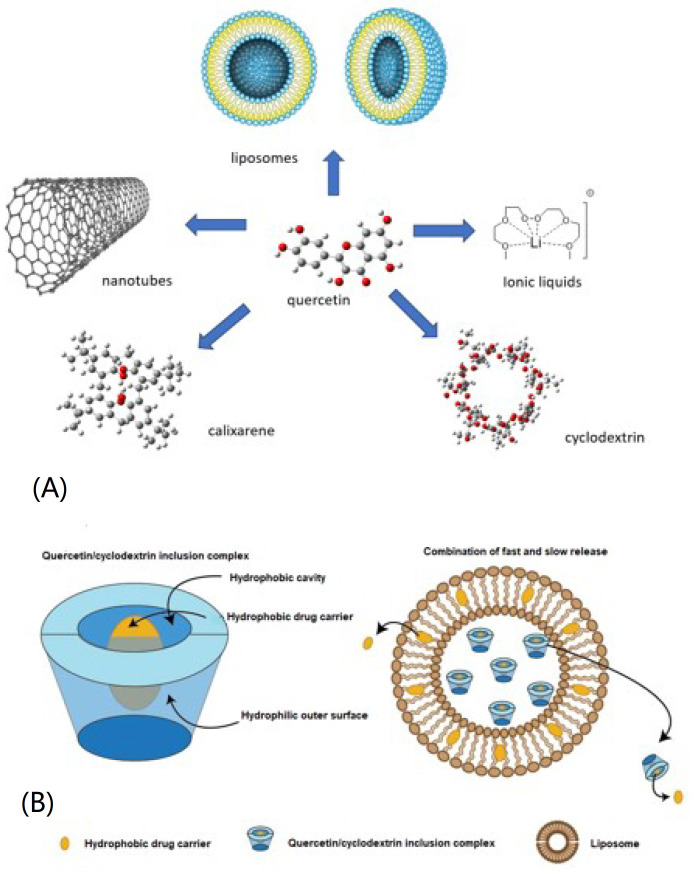
Future formulations of quercetin in various macromolecules sketched in PowerPoint installed on Windows 10. Quercetin can be incorporated into various macromolecules such as nanotubes, fullerenes, etc., along with their combinations (**A**). In addition, quercetin can be incorporated into liposomes in a simple form or complexed in CDs or calixarenes or other forms in which slow or fast release can be controlled (**B**).

In conclusion, this review delves into the diverse biological and medicinal implications of quercetin, especially its anti-inflammatory, antioxidant, anti-tumor, and antiviral properties. The exploration of quercetin′s synergistic potential when combined with other drugs and natural products holds promise for the development of innovative medications. Such combinations not only offer the prospect of novel drugs but also present an avenue for mitigating side effects and toxicity. Addressing the challenge of quercetin′s poor bioavailability, researchers have successfully conducted complexation within various macromolecules, including cyclodextrins, polymers, liposomes, and nanomaterials. Both experimental and computational efforts have resulted in stable complexes, demonstrating enhanced bioavailability in vitro. Future applications may involve quercetin complexes with cyclodextrins inside the liposomes, suggesting a wide spectrum of medicinal possibilities (Scheme 12A). In addition, combined macromolecules like cyclodextrins and calixarenes can be used to engulf quercetin (Scheme 12B). All these suggested future studies will designate the capability of quercetin to serve as a lead compound.

## Figures and Tables

**Table 1:** Examples of quercetin in combination with other compounds in medicinal chemistry.

Additive	Treatment of Disease
Vitamin C, vitamin B3, folic acid	Sickle cell disease (SCD) [114,115]
Catechin	Synergistic inhibition of the platelet function and dietary use [116]
Kaempferol	Prevention and treatment of hereditary cardiomyopathy [117]
Astragalin	Treatment of atopic dermatitits [118]
Statins	Reducing cholesterol levels [119]
Doxorubicin	Inhibiting liver cancer [120]
Oleuropein	Preventing and treating joint disorders [121]
Ibudilast	Treatment of Fragile X Syndrome [122]
Zafirlukast	Treating amyotrophic lateral sclerosis [123]
Rutin	Treating elevated blood lipid level-related diseases [124]
Polyphosphate	Treating osteoporosis [125]
Icaritin	Treatment of liver disease [126]
Vitamin D, retinol, and genistein	Improvement of skin conditions [127,128,129]
Maleic anhydride derivatives	Treatment of hepatocellular carcinoma [130]
Haloperidol	Releaving neuropathic pain [131]
Metformin	Preventing against immune diseases [132]
Luteonil and delphinidin	Treatment of endometriosis [133]
Myrecetin	Curing adenocarcinoma, prostate carcinoma, and breast cancer [134]

**Table 2:** Overall table of techniques that were used for the encapsulation of quercetin in macromolecules [156].

Technique	Reason That It Was Used
NMR spectroscopy	Structure elucidation of quercetin with cyclodextrins and observation of their complexation [91,157]
2D-DOSY NMR spectroscopy	Evaluation of the complex formation of quercetin with cyclodextrins [92,158]
Induced Fit Docking (IFD)	Evaluation of how effectively quercetin binds to essential viral components or enzymes. For instance, quercetin was used for IFD against acetylcholinesterase and butyrelcholinesterase [159,160,161,162].
Molecular dynamics	To obtain a deeper understanding of the stability and molecular interactions within the complexes formed by the “protein-ligand” pairs identified in the docking studies [92,163,164,165,166,167]
Molecular Mechanics Generalized Born Surface Area (MM GBSA)	To highlight the strongest binding capability of quercetin against different macromolecules [92,168]
Differential Scanning Calorimetry (DSC)	Validation of the formation of the complexes. For example, it was shown that quercetin was well distributed in the polyvinylpyrrolidone (PVP) matrix [169,170,171,172,173].
Fluorescence spectroscopic studies	Investigation of the interactions between quercetin and macromolecules. In particular, it was used in the formation of the dimeric assemblies of quercetin with cyclodextrins [91,174,175,176,177,178,179,180].
Solubility studies	Examination of the solubility of quercetin inside macromolecules in different pHs [155,181,182]
High-performance liquid chromatography (HPLC)	Validation of the purity and identification of the components. It was used for the determination of quercetin in herbal extracts [183,184,185,186].
Gas chromatography (GC)	Analysis of quercetin and its separation from different plants, materials, etc. [187,188,189]
UV/Vis spectroscopy	Quantification of quercetin in various contexts, encompassing pharmaceutical formulations [190], medicinal plants, beverages [191,192], and food.
Thin-layer chromatography (TLC)	Separation of quercetin from other flavonoids in a shared matrix [193,194,195,196]
Electrophoresis	Analysis of quercetin [197,198,199,200,201]
Cyclic voltammetry (CV)	Determination of the antioxidant activity of quercetin in lyophilized onion tissue of onion var [202,203,204]
Pulse voltammetry (DPV)	Determination of the antioxidant activity and the electrochemical parameters of quercetin [205]
Raman spectroscopy	Quantitative analysis of quercetin in onion peels [206,207,208,209,210]
Limit of detection (LOD) and limit of quantitation (LOQ)	Validation of the analytical method by determining quercetin in green tea [211]
Transmission Electron Microscopy (TEM)	Details for structural properties of quercetin in oil-in-water nanoemulsions [212,213]
Central Composite Design (CCD)	Evaluation of the effects of pH in determining quercetin in the presence of electroactive tannic acid [214,215]
Rheological measurements	Evaluation of the strength of the structure of quercetin with nanostructured lipid carriers in linseed oil [216]
Liquid Chromatography-Mass Spectroscopy (LC-MS)	Identification and quantification of quercetin in human hepatocytes as in vitro cell models [217]
Fourier-Transform Infrared Spectroscopy (FT-IR)	Analyzing the infrared absorption or emission of the molecule in buckwheat samples [218,219]
Capillary electrophoresis (CE)	Analysis of quercetin based on its electrophoretic mobility in red and white wine samples [197,220]
Enzyme-Linked Immunosorbent Assay (ELISA)	Quantitative analysis of quercetin to determine its anti-inflammatory effects in lipopolysaccharide stimulated cells [221,222,223]
Supercritical Fluid Chromatography (SFC)	Separation and extraction of quercetin from sumac fruits [224,225]
Flow Injection Analysis (FIA)	Subsequent detection of quercetin using normal and hot platinum microelectrode, showing the utility of Baranski’s method [226,227]
Solid-Phase Microextraction (SPME)	Extraction and analysis of quercetin, combined with HPLC-UV detection method, in green and black tea and coffee samples [184,228,229]
X-ray crystallography	Determination of the three-dimensional structure of quercetin crystals existing as hydrogen-bonded dimers, contributing to its unique biological activities [230]
Matrix-Assisted Laser Desorption/Ionization Mass Spectrometry (MALDI-MS)	Analysis of quercetin utilizing MIL-101(Cr) as surface-assisted matrix for replacing traditional organic matrices [231,232,233,234]
Supercritical Fluid Extraction (SFE)	Extraction of quercetin from Hyperici herba [224,235]
Solid-Phase Extraction (SPE)	Preparation of samples for extracting and determining quercetin’s and quercetin glucosides’ concentration in food products [236,237,238,239,240]

## Data Availability

Not applicable.
